# The molecular basis for allelic differences suggests *Restorer-of-fertility 1* is a complex locus in sugar beet (*Beta vulgaris* L.)

**DOI:** 10.1186/s12870-020-02721-9

**Published:** 2020-11-03

**Authors:** Takumi Arakawa, Muneyuki Matsunaga, Katsunori Matsui, Kanna Itoh, Yosuke Kuroda, Hiroaki Matsuhira, Kazuyoshi Kitazaki, Tomohiko Kubo

**Affiliations:** 1 Research Faculty of Agriculture, Hokkaido University, Kita-9, Nishi-9, Kita-ku, Sapporo, 060-8589 Japan; 2 Gifu Prefectural Research Institute for Agricultural Technology in Hilly and Mountainous Areas, Nakatsugawa, 508-0203 Japan; 3 Hokkaido Agricultural Research Center, National Agriculture and Food Research Organization, Shinsei Minami 9-4, Memuro, 082-0081 Japan

**Keywords:** Cytoplasmic male sterility, Nuclear-mitochondrial interaction, Hybrid breeding, *Oma1*, Allelic diversity, Plant reproduction

## Abstract

**Background:**

Cytoplasmic male sterility (CMS) is a widely used trait for hybrid seed production in many crops. Sugar beet CMS is associated with a unique mitochondrial protein named preSATP6 that forms a 250-kDa complex. *Restorer-of-fertility 1* (*Rf1*) is a nuclear gene that suppresses CMS and is, hence, one of the targets of sugar beet breeding. *Rf1* has dominant, semi-dominant and recessive alleles, suggesting that it may be a multi-allelic locus; however, the molecular basis for differences in genetic action is obscure. Molecular cloning of *Rf1* revealed a gene (*orf20*) whose protein products produced in transgenics can bind with preSATP6 to generate a novel 200-kDa complex. The complex is also detected in fertility-restored anthers concomitant with a decrease in the amount of the 250-kDa complex. Molecular diversity of the *Rf1* locus involves organizational diversity of a gene cluster composed of *orf20*-like genes (*RF-Oma1*s). We examined the possibility that members of the clustered *RF-Oma1* in this locus could be associated with fertility restoration.

**Results:**

Six yet uncharacterized *RF-Oma1*s from dominant and recessive alleles were examined to determine whether they could generate the 200-kDa complex. Analyses of transgenic calli revealed that three *RF-Oma1*s from a dominant allele could generate the 200-kDa complex, suggesting that clustered *RF-Oma1*s in the dominant allele can participate in fertility restoration. None of the three copies from two recessive alleles was 200-kDa generative. The absence of this ability was confirmed by analyzing mitochondrial complexes in anthers of plants having these recessive alleles. Together with our previous data, we designed a set of PCR primers specific to the 200-kDa generative *RF-Oma1*s. The amount of mRNA measured by this primer set inversely correlated with the amount of the 250-kDa complex in anthers and positively correlated with the strength of the *Rf1* alleles.

**Conclusions:**

Fertility restoration by sugar beet *Rf1* can involve multiple *RF-Oma1*s clustered in the locus, implying that stacking 200-kDa generative copies in the locus strengthens the efficacy, whereas the absence of 200-kDa generative copies in the locus makes the allele recessive irrespective of the copy number. We propose that sugar beet *Rf1* is a complex locus.

**Supplementary Information:**

**Supplementary information** accompanies this paper at 10.1186/s12870-020-02721-9.

## Background

Cytoplasmic male sterility (CMS) is a mitochondrial-encoded trait that is a prerequisite for hybrid seed production in some crop species [1–4]. CMS has often been associated with specific proteins in male-sterility inducing mitochondria, but the primary structures of these proteins differ among species [5, 6]. Some CMS-specific proteins, however, share several features such as having a hydrophobic domain [7]. In some crops such as maize, rapeseed with radish CMS, and sugar beet, CMS-specific proteins have been found in the mitochondrial membrane as oligomer forms [8–10].

CMS is suppressed by a nuclear gene termed *Restorer-of-fertility* (*Rf*) [11]. Usually, a dominant *Rf* allele suppresses CMS expression. Hence, seed parents of hybrid seed production should be homozygous recessive. On the other hand, when the F_1_ hybrid is meant for producing seed or fruit, the F_1_ plant should be fertility restored to secure pollination. As such, a discriminating *Rf* genotype is quite important for hybrid breeding, but *Rf*- and *rf* plants are phenotypically indistinguishable when they are combined with non-sterility inducing mitochondria. Therefore, uncovering the molecular basis for allelic differences in *Rf* will be a great help toward advancing hybrid breeding.

Molecular cloning of *Rf* revealed that its gene product is variable, but the most prominent class encodes pentatrico peptide repeat (PPR) proteins that are involved in post-transcriptional processing of mitochondrial genes [12–14]. PPR genes constitute a large gene family in the plant genome, and PPR-type *Rf* belongs to a subclass termed *Rf*-PPR-like genes (*RFL*) [15]. PPR-type *Rf* tends to cluster with *RFL*: the organization of the *RFL*-containing gene cluster is varied among genetic resources [16–18]. The significance of such a variable gene cluster is, however, unclear. From the viewpoint of crop breeding, diagnosis of *Rf* alleles is important. An unanswered question at present is whether the dominant/recessive nature of an *Rf* allele depends solely on the presence/absence of a specific gene copy in the cluster; in other words, whether the rest of the *Rf*-like gene copies can be ignored for allelic diagnosis.

Sugar beet cultivars are hybrids derived from the use of CMS [19]. Sugar beet CMS was discovered by Owen [20] and has been associated with a specific 39 kDa mitochondrial protein that is encoded by an origin-unknown open reading frame (ORF), *preSatp6* [10]. Translation product of *preSatp6* can be found in all examined organs [10]. This protein is highly hydrophobic and is detected from a 250-kDa protein complex when mitochondria are lysed in a mild detergent such as digitonin [21].

The genetics of fertility restoration in sugar beet is complex in some cases [22], but one of the best characterized sugar beet *Rf*s is *Rf1* [23]. Molecular cloning of *Rf1* revealed a gene cluster whose members resemble *Oma1*, a yeast gene involved in mitochondrial quality control [23]. The *Oma1*-like genes in the *Rf1* locus (hereafter *RF-Oma1*) are non-canonical *Oma1* genes because another apparently orthologous *Oma1* gene exists in the sugar beet genome; *RF-Oma1* has likely evolved by gene duplication followed by neofunctionalization [24].

In a transgenic experiment, one of the *RF-Oma1* genes was shown to increase pollen fertility [23]. When this *RF-Oma1* copy is expressed in suspension cells of CMS sugar beet, its translation products can bind with preSATP6 protein to generate a novel 200-kDa protein complex, whereas an *RF-Oma1* from a recessive *rf1* allele has no such activity [21]. The 200-kDa complex was also detected in *Rf1* fertility-restored anthers, indicating that the appearance of the 200-kDa complex is a hallmark of molecular interaction between *RF-Oma1* and *preSatp6* [21]. Concomitant with the appearance of the 200-kDa complex in fertility-restored anthers, the amount of the 250-kDa complex is highly reduced, yet the total amount of monomeric preSATP6 protein is almost unchanged [21]. We interpreted this phenomenon to be an alteration of the higher-order structure of preSATP6 protein by a molecular chaperone-like activity exerted by *RF-Oma1* [21].

**Fig. 1:**
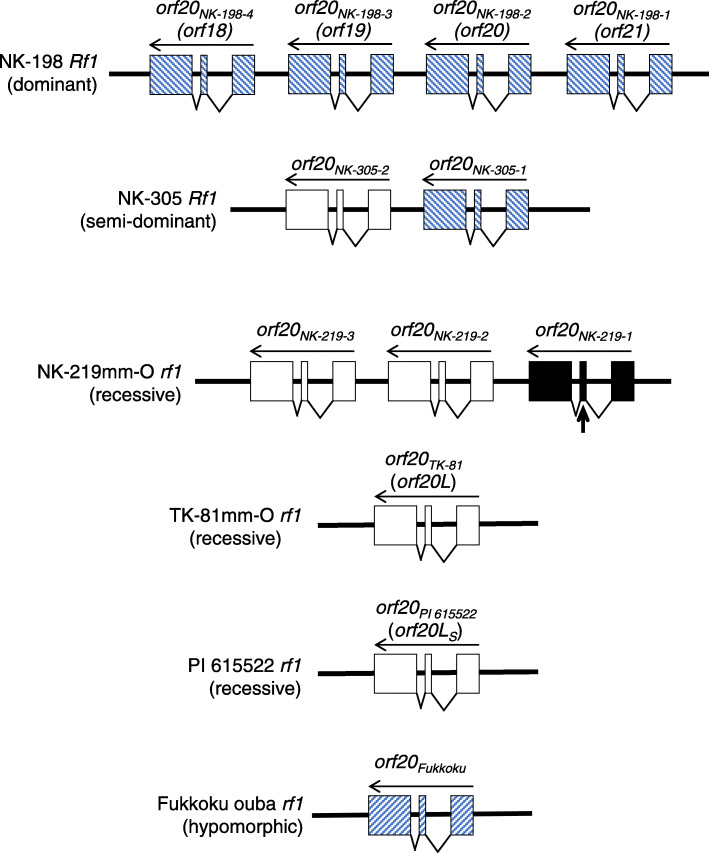
Organizational summary of beet *Rf1* loci. Names of alleles are shown at the left with their genetic action in parentheses. Boxes and wedges indicate exons and introns, respectively. Gene directions are from right to left. Names of individual *RF-Oma1* are shown above the genes with the old names used in previous papers in parentheses. The 200-kDa generative copies are striped; non-generative copies are open; and a pseudogene is filled. A vertical arrow indicates the position of a premature stop codon. This study characterized *orf20*_*NK-198-4*_, *orf20*_*NK-198-3*_, *orf20*_*NK-198-1*_, *orf20*_*NK-219-3*_, *orf20*_*NK-219-2*_ and *orf20*_*PI 615522*_. Sources of other information about these loci are [23, 25–27]

Our particular interest is the molecular diversity within the *Rf1* locus. To date, we have determined the nucleotide sequences of six *Rf1/rf1* alleles that differ in *RF-Oma1* copy number (Fig. 1) (for amino acid sequence homologies among these genes, see Additional file 1: Table S1). One of these alleles is a semi-dominant NK-305 *Rf1* whose homozygote is fully fertile, whereas the heterozygote is semi-fertile [25]. This genetic action contrasts with a dominant NK-198 *Rf1* whose heterozygote is fully fertile in the same condition, making us to infer that *Rf1* alleles in beet genetic resources have diverged in functionally [25]. The molecular organization of *Rf1* seems to have diverged significantly [26, 28, 29] with many of these alleles yet uncharacterized; hence, other alleles with different genetic actions are possible. Determining the molecular basis for differences in genetic action is necessary for evaluating genetic resources to find novel *Rf1* alleles.

Before the present study, six out of eleven *RF-Oma1* copies in Fig. 1 were uncharacterized (*orf20*_*NK-219-1*_ is an apparent pseudogene). This lack of information prompted us to investigate *Rf1* diversity by completely characterizing all of the *RF-Oma1* genes in Fig. 1. To our surprise, the dominant NK-198 *Rf1* is composed of four 200-kDa generative *RF-Oma1* copies. This finding caused us to propose the possibility that a specific *RF-Oma1* copy may not be the determinant of the allele’s nature, but rather the total amount of mRNA associated with the generation of the 200-kDa complex is one of the keys for defining the strength of the allele. In fact, we found an inverse correlation between the amount of mRNA and the amount of the 250-kDa complex. The amount of mRNA well explains the difference in the allele’s strength. Our finding implies that an increase in the number of 200-kDa generative *RF-Oma1* copies strengthens the allele, whereas non-generative *RF-Oma1* copies provide nothing to the allele for fertility restoration. We propose that sugar beet *Rf1* may be a complex locus whose action is determined by clustered *RF-Oma1* copies.

## Results

### A dominant *Rf1* allele decreases the accumulation of the 250-kDa protein complex in a gene-dose dependent manner

**Fig. 2:**
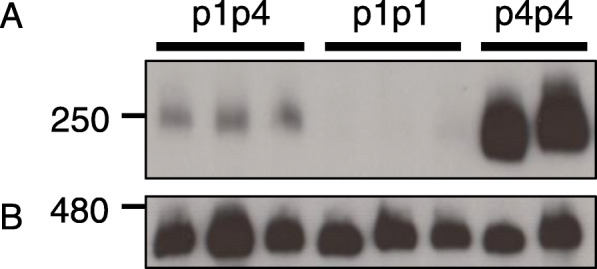
Immunoblot analysis of total cellular proteins from immature anthers collected from a BC_2_F_2_ population derived from TA-33BB-CMS x NK-198. Protein samples were electrophoresed in Blue Native polyacrylamide gels (4–16%). Size markers (kDa) are shown on the left. DNA marker types are shown at the top; genotypes p1p4 and p1p1 are heterozygous and homozygous forms of NK-198 *Rf1*, respectively. Genotype p4p4 is a homozygous recessive (i.e. *rf1rf1*). Immunoblots were probed with anti-preSATP6 (**a**) or anti-COXI (**b**)

During the course of our genetic analysis, we examined whether a dominant NK-198 *Rf1* allele had a gene-dosage effect on the amount of the 250-kDa protein complex. We used a BC_2_F_2_ population derived from a cross between TA-33BB-CMS (a CMS line) and NK-198 (the donor line of NK-198 *Rf1*) to select homozygotes and heterozygotes of NK-198 *Rf1*. Although both genotypes were phenotypically indistinguishable as they were fully fertile in our greenhouse [25], it was possible to diagnose the *Rf1* genotype by using the s17 DNA marker [25]. NK-198 *Rf1* is identified by a specific PCR band pattern named p1, whereas the *rf1* from TA-33BB-CMS (the same as TK-81 mm-O *rf1* in Fig. 1) is identified by p4. We selected homozygotes and heterozygotes of NK-198 *Rf1* and collected their immature anthers. Total anther cellular proteins were prepared in a buffer containing digitonin and electrophoresed on Blue Native (BN) polyacrylamide gels to detect protein complexes. Immunoblot analysis with anti-preSATP6 used conditions where the signal intensity of the 250-kDa complex could be quantified; sample amount, concentration of primary antisera and exposure time were adjusted as reported in [25]. Compared to the 250-kDa signal bands of *rf1rf1* from the same population, those of the heterozygotes were highly reduced but the faint bands could be seen (Fig. 2a); whereas, the 250-kDa signal bands of the homozygotes were almost invisible (Fig. 2a). It is unlikely that this decrease in the 250-kDa complex was due to an insufficient amount of loaded sample or inappropriate sample preparation because the levels of another mitochondrial complex detected by anti-COXI were comparable among the samples (Fig. 2b). These results suggested that NK-198 *Rf1* exerts a gene-dosage effect on the accumulation of the 250-kDa complex, although we were unaware of such a cumulative effect on the phenotype.

### All *RF-Oma1* copies in NK-198 *Rf1* have the potential to generate 200-kDa complexes

**Fig. 3:**
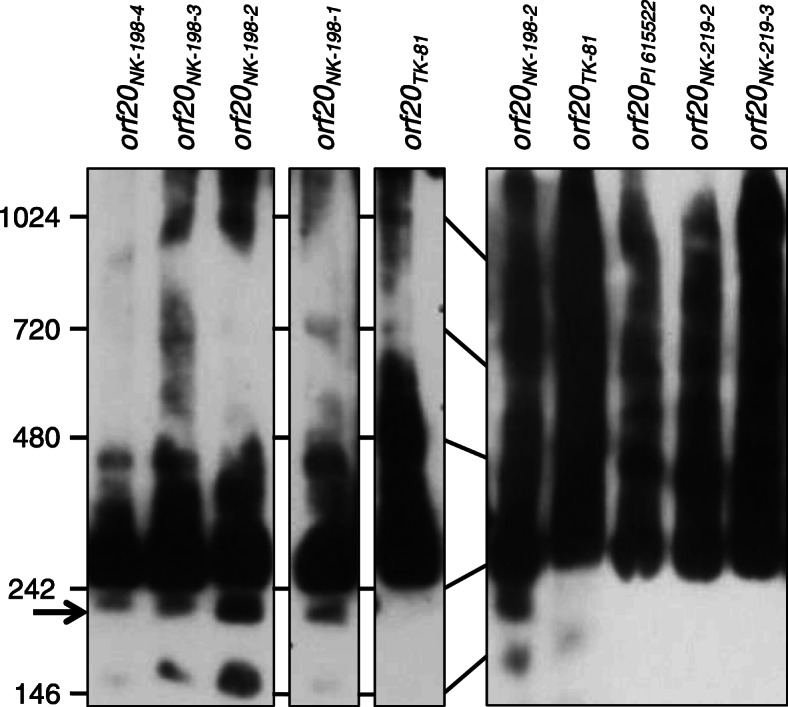
Immunoblot analysis of crude mitochondrial proteins extracted from transgenic cell lines. Protein samples were electrophoresed in Blue Native polyacrylamide gels (4–16%). Size markers (kDa) are shown on the left. FLAG-fused construct names are shown at the top. An arrow indicates the location of a 200-kDa band that is the hallmark of post-translational interaction with preSATP6

We further investigated the NK-198 *Rf1* allele at the molecular level to determine how such a large genetic effect on fertility restoration was exerted. According to Matsuhira et al. [23], NK-198 *Rf1* is comprised of four *RF-Oma1* copies, *orf18* to *orf21* (hereafter termed *orf20*_*NK-198-4*_ to *orf20*_*NK-198-1*_, respectively; see Fig. 1). Kitazaki et al. [21] showed that the translation products of *orf20*_*NK-198–2*_ (formerly *orf20*) have the ability to bind to preSATP6 protein and to generate a unique 200-kDa protein complex on BN polyacrylamide gels; however, the other *RF-Oma1* copies remained uncharacterized. In this study, we investigated whether the three uncharacterized *RF-Oma1* copies have the same potential for generating the 200-kDa protein complex as *orf20*_*NK-198–2*_. Each of the three copies (*orf20*_*NK-198-1*_, *orf20*_*NK-198-3*_, and *orf20*_*NK-198-4*_) was fused to a FLAG tag and regulated by the Cauliflower Mosaic Virus (CaMV) 35S promoter in binary vectors. The transgenes were introduced into CMS sugar beet suspension cells. Mitochondrial proteins from the transgenic cells were separated by BN-PAGE and probed with anti-preSATP6 to identify selectively the mitochondrial protein complex containing preSATP6. Smeared images were detected on the immunoblots, as was seen in previous studies (e.g. [25]). To our surprise, signal bands of 200-kDa were seen in the lanes of all four samples of *RF-Oma1* derived from NK-198 *Rf1* (Fig. 3), suggesting that all four sequences produce a protein that can interact with preSATP6 protein.

Although Matsuhira et al. [23] suggested that all *RF-Oma1* copies in NK-198 *Rf1* were expressed, the relative transcript levels produced by the four sequences were unknown. The largest obstacle for quantifying transcript abundance was the high sequence similarity among the four *RF-Oma1* copies, which precluded us from designing primer pairs to specifically quantify each of the four *RF-Oma1* mRNA species by PCR. Comparing the four *RF-Oma1* sequences, we noticed small insertions/deletions (indels) and several single nucleotide polymorphisms (SNPs) that enabled us to infer the ratio of *RF-Oma1* mRNA species (Additional file 2: Figure S1). We focused on a 6-bp indel in exon 1 and two SNPs in the 3′ trailer (Additional file 2: Figure S1); the indel distinguishes two mRNA groups (*orf20*_*NK-198-1*_ / *orf20*_*NK-198-4*_ and *orf20*_*NK-198-2*_ / *orf20*_*NK-198-3*_), and the two SNPs distinguish between three mRNA groups (*orf20*_*NK-198-4*_, *orf20*_*NK-198-3*_, and *orf20*_*NK-198-1*_ / *orf20*_*NK-198-2*_). Therefore, transcriptome data from anthers expressing NK-198 *Rf1* should contain reads of *RF-Oma1* mRNA that can be divided into these groups.

A fertility-restored BC_6_F_1_ plant derived from a cross between TA-33BB-CMS and NK-198 was selfed to obtain the NK-198 *Rf1* homozygote, which the s17 marker should identify as p1p1. RNA samples were extracted from tetrad-stage anthers before we conducted RNA-seq analysis. Read counts are summarized in Additional file 3: Table S2. We estimated the ratios of transcripts derived from the four *RF-Oma1* of NK-198 *Rf1* (Table 1); for example, we obtained transcript ratios of groups such as *orf20*_*NK-198-1*_ */ orf20*_*NK-198-4*_. On the other hand, the ratio of *orf20*_*NK-198-4*_ transcripts in the *RF-Oma1* transcript pool of the NK-198 *Rf1* homozygote was determined by utilizing SNPs in the 3′ UTR (Table S2). Using these values, the ratio of *orf20*_*NK-198-1*_ transcripts was calculated. Although the obtained values were approximations, the most abundant transcript was likely *orf20*_*NK-198–2*_, followed by *orf20*_*NK-198-4*_. The expression levels of the two lesser-expressed genes, *orf20*_*NK-198-1*_ and *orf20*_*NK-198-3*_, were comparable.

**Table 1:** Ratios of four *RF-Oma1* mRNAs in anthers of NK-198 *Rf1* homozygotes

*RF-Oma1*	Relative expression ratio			
	N_1 ^1^	N_2 ^1^	N_3 ^1^	Mean ^2^
*orf20*_*NK-198-1*_	0.20	0.17	0.16	0.18 ^c^
*orf20*_*NK-198–2*_	0.39	0.41	0.38	0.39 ^a^
*orf20*_*NK-198-3*_	0.16	0.17	0.17	0.17 ^c^
*orf20*_*NK-198-4*_	0.25	0.25	0.29	0.26 ^b^

^1^Biological replicates

^2^Differences in the letters indicate significance at *p* < 0.05 using Tukey’s multiple comparison test

### Analysis of recessive alleles from different origins

We also examined whether the remaining three uncharacterized *RF-Oma1* copies (i.e. copies from NK-219 mm-O *rf1* and PI 615522 *rf1*) had the potential for generating the 200-kDa complex. In this study, we first selected two sugar beet lines, PI 518644 and PI 615522, that have *orf20*_*NK-219-1*_ to *orf20*_*NK-219-3*_ and *orf20*_*PI 615522*_, respectively [26]. PI 518644 and PI 615522 are registered as ‘O-type’, a specific genotype that lacks a restoring allele but has non-sterility inducing mitochondria. The two lines were crossed with TA-33BB-CMS. All F_1_ plants (sixteen from TA-33BB-CMS x PI 518644 and five from TA-33BB-CMS x PI 615522) were completely male sterile.

Expression of *RF-Oma1* in the F_1_ plants was examined by reverse transcription-quantitative PCR (RT-qPCR). Total cellular RNAs were extracted from anthers at the meiotic and tetrad stages. *RF-Oma1* mRNAs were simultaneously detected by the primers common to all the copies. The results are summarized in Table 2. Expression levels were generally higher at the meiotic stage than at the tetrad stage in every genotype. F_1_ plants of TA-33BB-CMS x PI 615522 expressed *RF-Oma1* at a level comparable to that of TA-33BB-CMS, whereas those of TA-33BB-CMS x PI 518644 were 1.8 to 2.6-times higher than that of TA-33BB-CMS (Table 2). The difference appeared to be associated with the copy number of *RF-Oma1* in the zygote (Table 2).

**Table 2:** Relative transcript abundance of *RF-Oma1* measured by RT-qPCR in male sterile plants with recessive *rf1* alleles from different origins (*n* = 2)

Line/ cross combination	Total copy number of *RF-Oma1* in zygote	Reference gene	Anther developmental stage	
			Meiosis	Tetrad
TA-33BB-CMS	2	*Actin*	0.32 ± 0.08 ^a^	0.17 ± 0.00
		*ef1α*	0.37 ± 0.06	0.23 ± 0.01
TA-33BB-CMS x PI 615522	2	*Actin*	0.35 ± 0.01	0.21 ± 0.02
		*ef1α*	0.35 ± 0.08	0.30 ± 0.05
TA-33BB-CMS x PI 518644	3 ^b^	*Actin*	0.59 ± 0.12	0.37 ± 0.03
		*ef1α*	0.68 ± 0.08	0.55 ± 0.15

^a^Mean ± SD

^b^Excluding *orf20*_*NK-219-1*_ because it is an apparent pseudogene

**Fig. 4:**
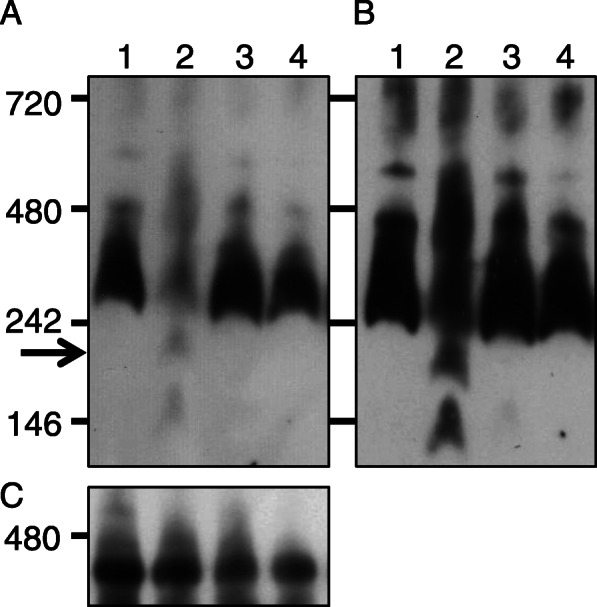
Immunoblot analysis of total cellular proteins from immature anthers collected from TA-33BB-CMS (lane 1), NK-198 (lane 2), TA-33BB-CMS x PI 615522 (lane 3) and TA-33BB-CMS x PI 518644 (lane 4). Protein samples were electrophoresed in Blue Native polyacrylamide gels (4–16%). Size markers (kDa) are shown on the left. An arrow indicates the 200-kDa band that is the hallmark of post-translational interaction with preSATP6. Immunoblots were probed with anti-preSATP6 (**a** and **b**) or anti-COXI (**c**). Exposure times to X-ray film were 10 s and 1 min for **a** and **b**, respectively

We tested whether any of the *RF-Oma1* translation products in PI 615522 or PI 518644 was 200-kDa generative. Total cellular proteins of immature anthers collected from F_1_ plants were subjected to BN-PAGE. Immunoblot analysis using anti-preSATP6 revealed that the two F1 plants gave images similar to that of TA-33BB-CMS. No 200-kDa signal band was detected even after prolonged exposures (Fig. 4).

We constructed FLAG-fused *orf20*_*NK-219-1*_, *orf20*_*NK-219-2*_ and *orf20*_*PI 615522*_ controlled by the CaMV 35S promoter in binary vectors. Mitochondrial proteins of the transgenic suspension cells were subjected to immunoblot analysis, and neither cell line showed the 200-kDa signal band on BN-PAGE (Fig. 3). In summary, none of the *RF-Oma1* copies identified to date in recessive alleles was found to be 200-kDa generative (summarized in Fig. 1).

### The 200-kDa generative mRNA quantity can explain the genetic action of the *Rf1* allele

Having detected the *RF-Oma1* mRNA of two recessive alleles and shown that the mRNA quantity appeared to correlate with the copy number of *RF-Oma1*, counting such *RF-Oma1* in the assessment of *Rf1* strength seemed to be inappropriate. We hypothesized that the genetic action of *Rf1* alleles is dependent on the sum of the *RF-Oma1* transcripts that have the potential to generate 200-kDa complexes. To design specific primers to such *RF-Oma1* copies, the nucleotide sequences of *RF-Oma1* shown in Fig. 1 were aligned to find SNPs or indels specific to forms that can generate 200-kDa complexes (Additional file 4: Figure S2). We found a 3-bp indel that discriminates 200-kDa generative copies from nongenerative ones, and a primer set was designed to specifically amplify the 200-kDa generative *RF-Oma1*. We tested its specificity by using binary vectors each having *RF-Oma1* as a template. As shown in Figure S3 (Additional file 5), PCR amplicons of the expected size appeared from 200-kDa generating *RF-Oma1* but not from non-generating forms.

Using this primer set, we quantified the mRNA of *RF-Oma1* in anthers. The homozygotes and heterozygotes of NK-198 *Rf1* were selected from the BC_6_F_2_ population. We selected the homozygotes and heterozygotes of NK-305 *Rf1* from a segregating population mentioned in [25]. In this population, NK-305 *Rf1* was marked by a specific pattern [p2] of the s17 DNA marker. Total cellular RNA was extracted from anthers at the meiotic and tetrad stages. The results of RT-qPCR are summarized in Table 3. In general, mRNA accumulation was similar between the two developmental stages. The amount of mRNA was highest in the NK-198 *Rf1* homozygote, followed by the NK-198 *Rf1* heterozygote, the NK-305 *Rf1* homozygote, and the NK-305 *Rf1* heterozygote, in that order. No mRNA was detected from *rf1rf1* when using this primer set (Table 3).

**Table 3:** Relative transcript abundance of *RF-Oma1* measured by RT-qPCR using common indel characteristics of 200-kDa generative copies [*n* = 2 (*ef1α* of p2p2) or *n* = 3 (the others)]

s17 marker type (genotype)	Reference gene	Anther developmental stage	
		Meiosis	Tetrad
p1p4 (NK-198 *Rf1* heterozygous)	*Actin*	0.43 ± 0.12 ^a^	0.39 ± 0.02
	*ef1α*	0.48 ± 0.14	0.48 ± 0.04
p1p1 (NK-198 *Rf1* homozygous)	*Actin*	0.86 ± 0.21	0.76 ± 0.18
	*ef1α*	1.00 ± 0.16	1.05 ± 0.30
p2p4 (NK-305 *Rf1* heterozygous)	*Actin*	0.16 ± 0.02	0.15 ± 0.01
	*ef1α*	0.20 ± 0.01	0.18 ± 0.06
p2p2 (NK-305 *Rf1* homozygous)	*Actin*	0.28 ± 0.03	0.34 ± 0.01
	*ef1α*	0.30 ± 0.04	0.42 ± 0.03
p4p4 (*rf1rf1*)	*Actin*	Not detected	Not detected
	*ef1α*	Not detected	Not detected

^a^Mean ± SD

We sought another value that correlated with the allelic strength of *Rf1*. Due to technical difficulties, we were unable to quantify the 200-kDa complex. Instead, we placed our focus on the degree to which the 250-kDa signal intensity was reduced by an *Rf1* allele (or two *Rf1* alleles) on BN-PAGE compared with *rf1rf1* (hereafter we refer to this value as Δ^250kDa^). We had previously shown that the decrease in the amount of the 250-kDa complex is likely caused by an alteration of higher-order structure of preSATP6 because the amount of monomeric preSATP6 is almost unchanged [21], whereas the amount of 420-kDa complex detected by anti-COXI is apparently unaffected [21]. Accumulation of monomeric COXI polypeptide appeared to be comparable between different genotypes [10]. For the NK-198 *Rf1*, the signal intensity of the 250-kDa signal band in Fig. 2 was normalized with that of the 420-kDa signal band detected by anti-COXI (Table 4). The signal intensity ratio for p1p4 (0.17 ± 0.02) was reduced by 1.57 compared with p4p4 (1.74 ± 0.26), hence the Δ^250kDa^ by a single NK-198 *Rf1* was 1.57. Note that the difference between p4p4 and p1p1 was 1.74, the upper limit of detection by this system.

**Table 4:** Amount of 250-kDa protein complex in anthers of different genotypes ^a^

s17 marker type (genotype)	250-kDa/420-kDa ratio	Difference from *rf1rf1* in the same population (Δ^250kDa^)
p4p4 (*rf1rf1*)	1.74 ± 0.26	–
p1p4 (NK-198 *Rf1* heterozygous)	0.17 ± 0.02	1.57
p1p1 (NK-198 *Rf1* homozygous)	Not detected	1.74

^a^The amount of the 250-kDa complex was estimated by the ratio of the signal intensity between the 250-kDa signal band detected by anti-preSATP6 and the 420-kDa signal band detected by anti-COXI

Arakawa et al. [25] estimated the accumulation of the 250-kDa complex in the homozygotes and heterozygotes of NK-305 *Rf1* and *rf1rf1* by the same procedure as our study. According to [25], the 250-kDa/420-kDa ratios were 1.57 ± 0.16 and 0.86 ± 0.07 for *rf1rf1* and NK-305 heterozygous, respectively, hence the Δ^250kDa^ by a single NK-305 *Rf1* copy was 0.71. The ratio for the NK-305 *Rf1* homozygous was 0.12 ± 0.01, a value that differed from the *rf1rf1* by 1.45 or twice of the Δ^250kDa^ of single NK-305 *Rf1*.

**Fig. 5:**
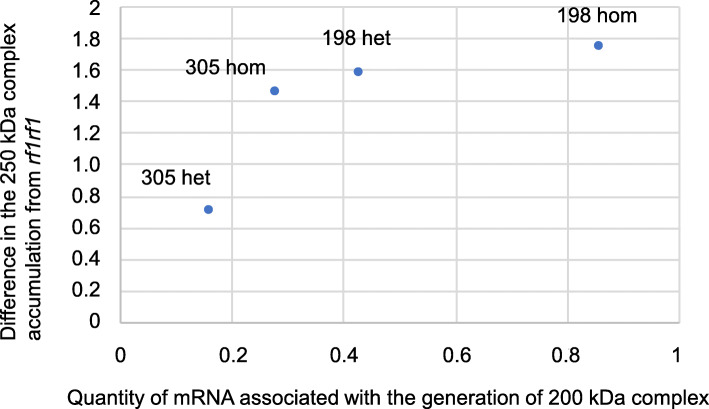
Relationship between the quantity of mRNA associated with the generation of the 200-kDa complex (horizontal axis) and the relative difference in 250-kDa complex accumulation compared to *rf1rf1* (Δ^250kDa^, vertical axis). Dots represent data from the NK-305 *Rf1* heterozygote (305 het), the NK-305 *Rf1* homozygote (305 hom), the NK-198 *Rf1* heterozygote (198 het), and the NK-198 *Rf1* homozygote (198 hom). Anthers were collected at the meiotic stage for RNA isolation, and the reference gene for RT-qPCR was *Actin*

The quantity of mRNA associated with 200-kDa generation and the Δ^250kDa^ were measured in the four genotypes (i.e. homozygous and heterozygous NK-305 *Rf1* and NK-198 *Rf1*). We plotted these data sets as shown in Fig. 5 and Figure S4 (Additional file 6) and found a positive relationship between the level of transcript accumulation and the Δ^250kDa^.

## Discussion

The 250-kDa protein complex containing the CMS-specific polypeptide preSATP6 is the target molecule of *Rf1* [21]. We were interested in the quantitative aspects of naturally occurring *Rf1* alleles. The molecular basis for the allelic differences in *Rf1* likely involves multiple *RF-Oma1* copies in the allele but not a specific copy.

A notable finding of this study is that all the *RF-Oma1* copies in NK-198 *Rf1* are capable of generating the 200-kDa complex. This finding was unexpected because transgenics expressing *orf20*_*NK-198-1*_, *orf20*_*NK-198-3*_ and *orf20*_*NK-198-4*_ were apparently male sterile [23]. Reconciliation between these differing results is possible if a hypomorphic *Rf1* allele is considered that encodes a single *RF-Oma1* capable of generating the 200-kDa complex but barely restores fertility due to the production of a small amount of mRNA [27]. This allele, Fukkoku-ouba *rf1* (see Fig. 1), is non-restoring but plants with this allele sometimes develop anther contents similar to semi-fertile plants (hence, we hesitate to call this allele recessive), as was seen in the three transgenics (our unpublished observations). According to Arakawa et al. [27] and this study, the amount of *orf20*_*Fukkoku*_ mRNA was inferred to be comparable to that in *orf20*_*NK-198-1*_, *orf20*_*NK-198-3*_ or *orf20*_*NK-198-4*_. It seems possible that each of the three *RF-Oma1* copies is functionally equivalent to Fukkoku ouba *rf1*; hence, transgenics expressing each of these constructs remained male sterile.

We had reported that transgenic sugar beet expressing *orf20*_*NK-198–2*_ was restored to semi-fertility despite the transgene being derived from a strong allele of NK-198 *Rf1* [23]. This result suggested the possibility that fertility restoration by *orf20*_*NK-198–2*_ explains only part of the total strength of NK-198 *Rf1*. This notion may be supported by the semi-fertile phenotype of the NK-305 *Rf1* heterozygote reported in [25], which is very similar to that of the *orf20*_*NK-198–2*_-expressing transgenics. According to Arakawa et al. [25], NK-305 *Rf1* is composed of *orf20*_*NK-305-1*_ and *orf20*_*NK-305-2*_, of which only the former is 200-kDa generative (see Fig. 1). Therefore, data from genotype p2p4 in Table 3 can be interpreted as those coming from *orf20*_*NK-305-1*_ mRNA. Based on the figures in Table 1, we estimate that *orf20*_*NK-198–2*_ mRNA accounts about 40% of p1p4 in Table 3. Accordingly, our results suggest that *orf20*_*NK-198–2*_ and *orf20*_*NK-305-1*_ generate comparable amounts of mRNA. Thus, the phenotypes of transgenic plants described in Matsuhira et al. [23] seem to be consistent with phenotypes expressed by a single NK-305 *Rf1* [25]. Altogether, transgenics expressing each of the single dissected *RF-Oma1* copies from NK-198 *Rf1* phenocopied the genetic action of other *Rf1* alleles, but none of them replicated the genetic action of NK-198 *Rf1*. Of course, we cannot exclude other possibilities such as insufficient expression of the transgene or a background effect are involved in the phenotypes of the transgenics.

Possibly, all four *RF-Oma1* in NK-198 *Rf1* participate in fertility restoration to achieve completely fertile plants. In this model, the principal restorer is *orf20*_*NK-198–2*_, but full restoration needs the other three *RF-Oma1* copies to provide a sufficient quantity of mRNA to reduce the accumulation of the 250-kDa complex to a level that allows normal pollen development. This model presumes a cumulative effect of *RF-Oma1* that can be inferred by the gene dose effect of NK-305 *Rf1*; its homozygotes were more fertility-restored than the heterozygotes [25]. A gene dose effect on the 250 kDa complex quantity was also obvious in NK-198 *Rf1*. This cumulative effect is also suggested by Table 3. We propose that the sugar beet *Rf1* locus may be a complex locus whose alleles are characterized by the composition of the clustered *RF-Oma1* copies.

The strength of the *Rf1* allele is represented by a reduction in the amount of the 250-kDa complex in anthers (i.e. Δ^250kDa^). We estimated the Δ^250kDa^ for a single NK-198 *Rf1* as 1.57 (Table 4), which is about twice that of a single NK-305 *Rf1* (0.71) (Table 4) and is consistent with the amount of 200-kDa generative mRNA (compare genotypes p1p4 and p2p4 in Table 3). Perhaps the amount of the 250-kDa complex is inversely correlated with the quantity of *RF-Oma1* mRNA associated with 200-kDa generation. In support of this hypothesis, we found that the amounts of mRNA and the Δ^250kDa^ were positively correlated (Fig. 5). This plot poses the notion that NK-198 *Rf1* is too efficient when in the homozygous condition as the amount of 200-kDa generative mRNA in the NK-198 *Rf1* homozygote is twice that of the heterozygote (Table 3), making its potential Δ^250kDa^ equal to 3.14 (2 × 1.57, see genotype p1p4 in Table 4). This potential Δ^250kDa^, however, cannot be fully directed to the 250-kDa complex because 1.74 (250-kDa accumulation in *rf1rf1*) is the upper limit, and the residual 1.40 remains unused. The residual activity would need to be managed if *RF-Oma1* has some side-effect that is harmful for the plant, otherwise the frequency of such alleles would decline by counter selection. Although this hypothesis may be related to the observation that the frequency of genotypes that restore full fertility are rare in sugar beet [30], further study is necessary to characterize the genetic diversity of *Rf1* in sugar beet with the aim of exploiting genetic resources to advance sugar beet breeding.

## Conclusions

Sugar beet *Rf1* includes alleles of different strengths, including dominant and semi-dominant alleles. The dominant NK-198 *Rf1* is composed of four copies of *RF-Oma1* that have the potential to generate the 200-kDa complex, whereas the semi-dominant NK-305 *Rf1* allele has one 200-kDa generative copy and one non-generative copy. *RF-Oma1* copies of recessive alleles have no such activity, but they are transcribed, and the amount of mRNA seems to be copy-number dependent. Using specific primer sets for the 200-kDa generative copies, the mRNA was quantified. The transcript abundance inversely correlated with the quantity of the 250-kDa protein complex composed of preSATP6, the CMS-specific mitochondrial protein. The mRNA quantity also explained the different genetic actions exemplified by NK-198 *Rf1* and NK-305 *Rf1*. We propose a hypothesis in which sugar beet *Rf1* is a complex locus with multiple alleles whose characters are determined by the function of the *RF-Oma1* copies clustered in the alleles. This hypothesis implies that none of the dissected *RF-Oma1* copies would be sufficient to restore complete fertility even though they are derived from a strong *Rf1* allele.

## Methods

### Plant materials

Beet (*Beta vulgaris ssp. vulgaris*) lines or accessions used or mentioned in this study are listed in Table 5. Sugar beet lines NK-198, NK-219 mm-CMS, NK-305, TA-33BB-CMS and TA-33BB-O were developed at the National Agriculture and Food Research Organization, Japan. NK-198 and NK-305 are fertility restored lines [21, 23, 25]. TA-33BB-CMS and TA-33BB-O have identical nuclear genotypes, but the former and the latter have male sterility-inducing and non-inducing mitochondria, respectively. The *RF-Oma1* sequences of the two lines are identical to *orf20*_*TK-81*_ [24]. NK-219 mm-CMS is a CMS line competent for *Agrobacterium*-mediated transformation [31]. PI 518644 and PI 615522 are U.S. sugar beet lines developed by U. S. Department of Agriculture [26]. ‘Fukkoku ouba’ is a Japanese leaf beet accession [27]. Crosses were done by using paper bags as described in [26]. Plants were grown in the greenhouse or the field at the Field Science Center for the Northern Biosphere, Hokkaido University. Pollen fertility was visually inspected and classified into fully normal, semi-fertile (anthers become orange in color but rarely dehisce), and completely male sterile as described in [25].

**Table 5:** Beet lines/accessions used or mentioned in this study

Line/accession	Cultivar type	Cytoplasm ^a^	Genotype	Origin
NK-198	Sugar beet	S	*Rf1Rf1*	NARO ^b^
NK-219 mm-CMS		S	*rf1rf1*	NARO
NK-305		S	*Rf1Rf1*	NARO
PI 518644		N	*rf1rf1*	NARO
PI 615522		N	*rf1rf1*	USDA ^c^
TA-33BB-CMS		S	*rf1rf1*	USDA
TA-33BB-O		N	*Rf1Rf1*	NARO
‘Fukkoku ouba’	Leaf beet	N	*rf1rf1*	NARO

^a^S and N denote male-sterility inducing cytoplasm and non-male sterility inducing cytoplasm, respectively

^b^National Agriculture and Food Research Organization, Japan

^c^U. S. Department of Agriculture

### Genotyping

DNA marker s17 was detailed previously [25, 27, 29]. Total cellular DNA was isolated from green leaves by the standard CTAB-based method [32]. The nucleotide sequences of PCR primers are shown in Table S3 (Additional file 7).

### Protein complex analysis

Protein complexes of anthers or crude mitochondria were separated by Blue Native polyacrylamide gel electrophoresis (BN-PAGE) according to [21]. A NativePAGE Novex BisTris Gel system (Thermo Fisher Scientific, Waltham, MA, USA) was used. Separated complexes were blotted onto a Hybond-P PVDF membrane (GE Healthcare, Little Chalfont, UK) according to the manufacturer’s instruction manual. Primary antisera were anti-preSATP6 [10], anti-COXI [10], and anti-FLAG (Medical and Biological Laboratories, Nagoya, Japan). Antisera were diluted as described in [25]. The secondary antibody was HRP-conjugated goat anti-mouse IgG and HRP-conjugated goat anti-rabbit IgG (Jackson ImmunoResearch, West Grove, PA, USA). Conditions for quantification were modified as previously described [25]. Uncropped images are provided in Additional file 8.

### Transgenic callus

Similar procedures to those in [24, 25, 27] were adopted to construct transgenes. Open reading frames (ORFs) of interest were PCR amplified from total cellular DNA as described in [23] and cloned into pDONR/zeo via the Gateway system (Thermo Fisher Scientific). A FLAG tag was fused by in vitro mutagenesis using a PrimeSTAR Mutagenesis Basal Kit (Takara Bio, Kusatsu, Japan). The resultant genes were transferred into pMDCΩ, a Gateway-compatible binary vector [21]. Transgenes were introduced into NK-219 mm-CMS callus via Agrobacterium LBA 4404 [31]. For the sequences of oligonucleotide primers, see Table S3 (Additional file 7).

### Reverse transcription-quantitative PCR

Anthers from the meiotic or tetrad stages were collected as described in [21]. An RNeasyPlant Mini Kit (Qiagen, Valencia, CA, USA) and RNase-free DNase I (Takara Bio) were used for sample preparation. Complementary DNA was synthesized with SuperScript III First-Strand Synthesis System (Thermo Fisher Scientific) and an oligo dT primer. Conditions for quantifying transcript levels were followed as described in [25, 27]. Primers for RT-qPCR are shown in Table S3 (Additional file 8).

### RNA-Seq

Total cellular RNA was isolated from tetrad stage anthers by using an RNeasy Plant Mini Kit (Qiagen, Valencia, CA, USA). RNA (two μg) was sent to Macrogen Corp. Japan (Kyoto, Japan) and then quality checked with a 2100 Bioanalyzer (Agilent Technologies, Palo Alto, Calif, USA). The libraries were prepared using a TruSeq Stranded mRNA LT Sample Prep Kit (Illumina, San Diego, CA, USA) and sequenced in pair-ends, 101 bp for each read by Novaseq6000 (Illumina). Raw sequence data were trimmed and quality checked by Sickle ver.1.33 (https://github.com/najoshi/sickle) at a quality threshold of Q20 and a length threshold of 50 bp and FastQC ver.0.11.7 (https://www.bioinformatics.babraham.ac.uk/projects/fastqc/), respectively. Reference sequences are shown in Figure S2 (Additional file 2) and correspond to DDBJ accession numbers AB646133 and AB646135. The filtered reads were aligned to reference sequences using HiSAT2 ver.2.1.0 (https://ccb.jhu.edu/software/hisat2/index.shtml). The mapped reads having no discrepancy with each reference sequence were visually checked and counted using IGV ver.2.4.13 (https://software.broadinstitute.org/software/igv/).

## Supplementary Information

**Additional file 1: Table S1.** Percent identity of amino-acid sequences among *RF-Oma1* shown in Fig. 1. Results of pairwise comparison of eleven *RF-Oma1* sequences are shown.

**Additional file 2: Figure S1.** Alignments of reference sequences derived from each *RF-Oma1* in NK-198 *Rf1*. Polymorphic sites of *RF-Oma1* in NK-198 *Rf1* are shown.

**Additional file 3: Table S2** Read-count and ratio of mapped reads on reference sequences. Read-count and ratio of *orf20*_*NK-198-1*_, *orf20*_*NK-198–2*_, *orf20*_*NK-198-3*_ and *orf20*_*NK-198-4*_ in anther.

**Additional file 4: Figure S2** Alignment of partial nucleotide sequences of *RF-Oma1* exon 1. Nucleotide sequences were aligned to design a primer set specific to 200-kDa generative class.

**Additional file 5: Figure S3**. Agarose gel electrophoresis of PCR products. Specificity of the primer set was tested.

**Additional file 6: Figure S4**. Scatter plot of quantity of mRNA associated with the generation of 200-kDa complex and difference in the 250-kDa complex accumulation from *rf1rf1*. Positive correlation between the amount of mRNA of 200 kDa generatives and Δ^250kDa^ in meiosis (*actin*) and tetrad (*actin* and *ef1α*) stages is shown.

**Additional file 7: Table S3** Nucleotide sequences of primers used in this study. Nucleotide sequences of primers used in this study are shown.

**Additional file 8.** Uncropped images. Uncropped images used for Figs. 2, 3 and 4 are shown.

## Data Availability

All data generated or analyzed during this study are included in this published article and its supplementary information files. Sequence data were deposited in the DDBJ Sequence Read Archive (DRA010937).

## Abbreviations

CMS: Cytoplasmic male sterility
PPR: Pentatrico peptide repeat
RFL: Restorer-of-fertility like
CaMV: Cauliflower Mosaic Virus
BN-PAGE: Blue-Native polyacrylamide gel electrophoresis
ORF: Open reading frame
RT-qPCR: Reverse transcription-quantitative polymerase chain reaction
