# Supplementary material for: The molecular basis for allelic differences suggests Restorer-of-fertility 1 is a complex locus in sugar beet (Beta vulgaris L.)
Source: BMC Plant Biol. 2020 Nov 3;20:503. doi: 10.1186/s12870-020-02721-9 (PMC7607634; doi:10.1186/s12870-020-02721-9)
Supplement: Supplementary file 2 — Additional file 2: Figure S1. Alignments of reference sequences derived from each RF-Oma1 in NK-198 Rf1. Polymorphic sites of RF-Oma1 in NK-198 Rf1 are shown. [file 12870_2020_2721_MOESM2_ESM.pdf]

A

```
Exon_orf20NK-198-1   CACTGGAAAG AGAGATTAAT CACCATGAAC TCGAACTCGA ACTCGAAAGA GATGAAACTT 557
Exon_orf20NK-198-2   CACTGGAAAG AGAGATTAAT CACCATGAAC TCGAACTCGA A------AGA GATGAAACTT 551
Exon_orf20NK-198-3   CACTGGAAAG AGAGATTAAT CACCATGAAC TCGAACTCGA A------AGA GATGAAACTT 542
Exon_orf20NK-198-4   CACTGGAAAG AGAGATTAAT CACCATGAAC TCGAACTCGA ACTCGAAAGA GATGAAACTT 557
         consensus   CACTGGAAAG AGAGATTAAT CACCATGAAC TCGAACTCGA ACTCGAAAGA GATGAAACTT

                                   ▼
Exon_orf20NK-198-1   TCAAGGAGAA AACCATTTGG AAGGAGGAGA CAGTTGATGA TAAAGATAGT A 609
Exon_orf20NK-198-2   TCAAGGAGAA AACCATTTGG AAGGAGGAGA CAGTTGATGA TAAAGATAGT A 603
Exon_orf20NK-198-3   TCAAGGAGAA AACCATTTGG AAGGAGGAGA CAGTTGATGA TAAAGATAGT A 594
Exon_orf20NK-198-4   TCAAGGAGAA AACCATTTGG AAGGAGGAGA CAGTTGATGA TAAAGATAGT A 609
         consensus   TCAAGGAGAA AACCATTTGG AAGGAGGAGA CAGTTGATGA TAAAGATAGT A
```

B

```
                                                                              ▼
3'UTR_orf20NK-198-1  CCATTTACCA ACCAGCATCT TCTTTTAGCA GCTTCGCCTG TTTATGAATT GTGGTAATCA 1926
3'UTR_orf20NK-198-2  CCATTTACCA ACCAGCATCT TCTTTTAGCA GCTTCGCCTG TTTATGAATT GTGGTAATCA 1920
3'UTR_orf20NK-198-3  CCATTTACCA ACCAGCATCT TCTTTTAGCA GCTTCGCCTG TTTATGAATT GTGGTAATCA 1943
3'UTR_orf20NK-198-4  CCATTTACCA ACCAGCATCT TCTTTTAGCA GCTTCGCCTG TTTATGAATT ATGGTAATCA 1926
          consensus  CCATTTACCA ACCAGCATCT TCTTTTAGCA GCTTCGCCTG TTTATGAATT *TGGTAATCA

3'UTR_orf20NK-198-1  AAATTAAACA GCTCATCGAT CATTATATTG TCGTTATATT TCATCTGTTT GACAAAGTTT 1986
3'UTR_orf20NK-198-2  AAATTAAACA GCTCATCGAT CATTATATTG TCGTTATATT TCATCTGTTT GACAAAGTTT 1980
3'UTR_orf20NK-198-3  AAATTAAACA GCTCATGGAT CATTATATTG TCGTTATATT TCATCTGTTT GACAAAGTTT 2003
3'UTR_orf20NK-198-4  AAATTAAACA GCTCATGGAT CATTATATTG TCGTTATATT TCGTCTGTTT GACAAAGTTT 1986
          consensus  AAATTAAACA GCTCAT*GAT CATTATATTG TCGTTATATT TC*TCTGTTT GACAAAGTTT
```

Fig. S1. Alignments of reference sequences derived from each *RF-Oma1* in NK-198 *Rf1*. Nucleotide residues are numbered from the from the translational initiation codon. Exon and 3' trailer regions are shown (A and B, respectively). Dashes indicate gaps inserted for maximum matching. The nucleotide positions for read-number counting are shown with black triangles.
