# Supplementary material for: The molecular basis for allelic differences suggests Restorer-of-fertility 1 is a complex locus in sugar beet (Beta vulgaris L.)
Source: BMC Plant Biol. 2020 Nov 3;20:503. doi: 10.1186/s12870-020-02721-9 (PMC7607634; doi:10.1186/s12870-020-02721-9)
Supplement: Supplementary file 4 — Additional file 4: Figure S2 Alignment of partial nucleotide sequences of RF-Oma1 exon 1. Nucleotide sequences were aligned to design a primer set specific to 200-kDa generative class. [file 12870_2020_2721_MOESM4_ESM.pdf]

```
              N  Q  S  T  N  K  C  S  G  L  F  G  S  A  K  S  G  Y  F  N
198-1        AATCAATCTACTAATAAGTGTAGTGGGTTATTTGGGTCTGCAAAATCTGGGTATTTTAAT 180
198-2        AATCAATCTACTAATAAGTGTAGTGGGTTATTTGGGTCTGCAAAATCTGGGTATTTTAAT 180
198-3        AATCAATCTACTAATAAGTGTAGTGGGTTATTTGGGTCTGCAAAATCTGGGTATTTTAAT 180
198-4        AATCAATCTACTAATAAGTGTAGTGGGTTATTTGGGTCTGCAAAATCTGGGTATTTTAAT 180
305-1        AATCAATCTACTAATAAGTGTAGTGGGTTATTTGGGTCTGCAAAATCTGGGTATTTTAAT 180
Fukkoku      AATCAATCTACTAATAAGTGTAGTGGGTTATTTGGGTCTGCAAAATCTGGGTATTTTAAT 180
219-2        AATCAATCTACTAA---GTGTAGTGGGTTATTTGGGTCTGCAAAATCTGGGTATTTTAAT 177
219-3        AATCAATCTACTAA---GTGTAGTGGGTTATTTGGGTCTGCAAAATCTGGGTATTTTAAT 177
305-2        AATCAATCTACTAA---GTGTAGTGGGTTATTTGGGTCTGCAAAATCTGGGTATTTTAAT 177
615522       AATCAATCTACTAA---GTGTAGTGGGTTATTTGGGTCTGCAAAATCTGGGTATTTTAAT 177
TK-81        AATCAATCTACTAA---GTGTAGTGGGTTATTTGGGTCTGCAAAATCTGGGTATTTTAAT 177
```

Fig. S2 Alignment of partial nucleotide sequences of *RF-Oma1* exon 1 from *orf20*$_{NK-198-1}$ (198-1), *orf20*$_{NK-198-2}$ (198-2), *orf20*$_{NK-198-3}$ (198-3), *orf20*$_{NK-198-4}$ (198-4), *orf20*$_{NK-305-1}$ (305-1), *orf20*$_{fukkoku}$ (Fukkoku), *orf20*$_{NK-219-2}$ (219-2), *orf20*$_{NK-219-3}$ (219-3), *orf20*$_{NK-305-2}$ (305-2), *orf20*$_{PI\ 615522}$ (615522), and *orf20*$_{TK-81}$ (TK-81). Dashes are incorporated for maximum matching. Nucleotide sequences are numbered from the first letters of initiation codons. Amino acid sequence deduced from nucleotide sequence is shown above. The common indels to those *RF1-Oma1* having ability to generate the 200-kDa protein complex are yellow-highlighted. Position of primer to discriminate those *RF-Oma1* is shown by a horizontal arrow.
