# Supplementary material for: The molecular basis for allelic differences suggests Restorer-of-fertility 1 is a complex locus in sugar beet (Beta vulgaris L.)
Source: BMC Plant Biol. 2020 Nov 3;20:503. doi: 10.1186/s12870-020-02721-9 (PMC7607634; doi:10.1186/s12870-020-02721-9)
Supplement: Supplementary file 5 — Additional file 5: Figure S3. Agarose gel electrophoresis of PCR products. Specificity of the primer set was tested. [file 12870_2020_2721_MOESM5_ESM.pptx]

## Slide 1
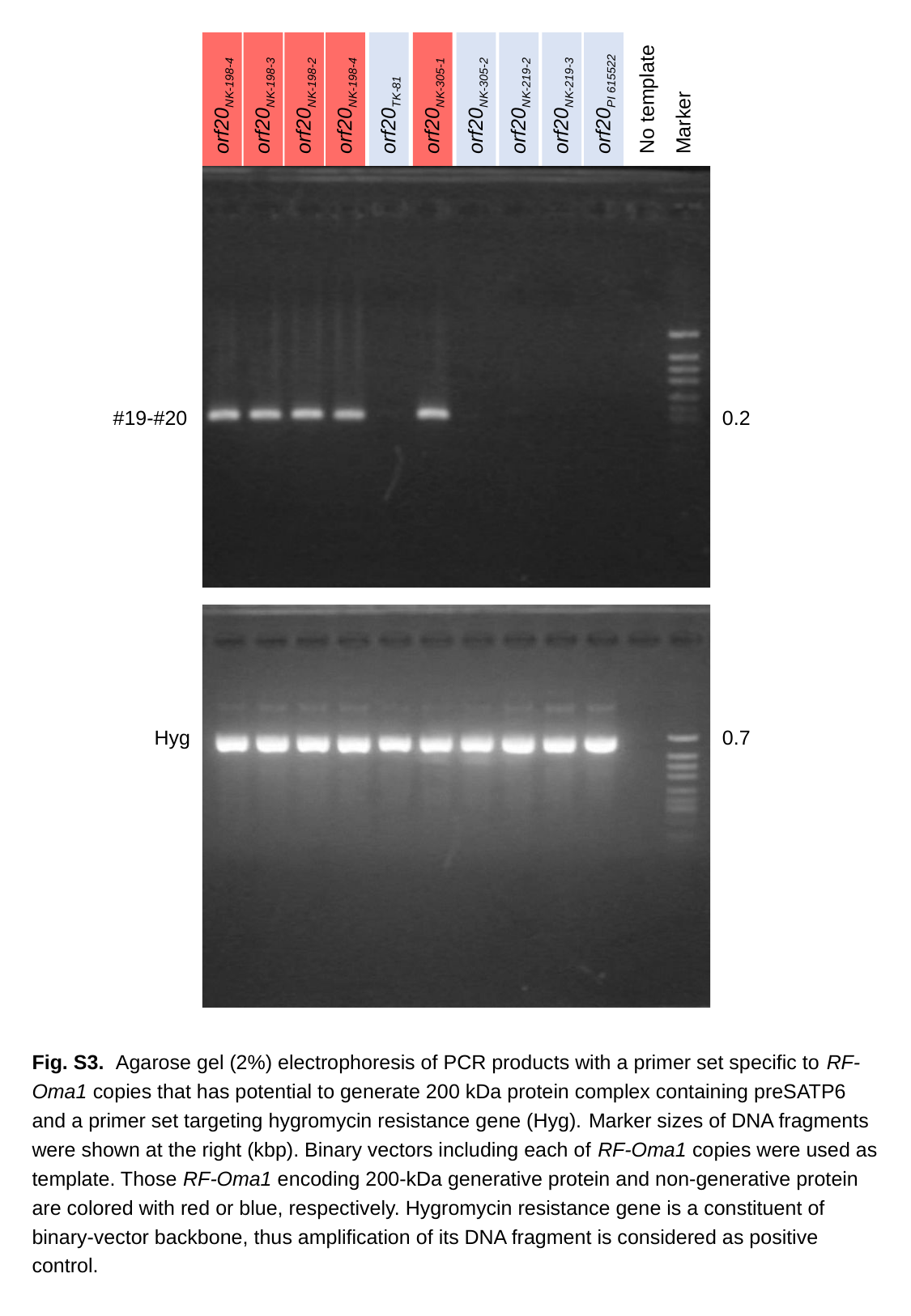

No template
Marker
orf20NK-198-4
orf20NK-198-3
orf20NK-198-4
orf20TK-81
orf20NK-305-1
orf20NK-305-2
orf20NK-219-2
orf20NK-219-3
orf20PI 615522
orf20NK-198-2
#19-#20
0.2
Hyg
0.7
Fig. S3. Agarose gel (2%) electrophoresis of PCR products with a primer set specific to RF-Oma1 copies that has potential to generate 200 kDa protein complex containing preSATP6 and a primer set targeting hygromycin resistance gene (Hyg). Marker sizes of DNA fragments were shown at the right (kbp). Binary vectors including each of RF-Oma1 copies were used as template. Those RF-Oma1 encoding 200-kDa generative protein and non-generative protein are colored with red or blue, respectively. Hygromycin resistance gene is a constituent of binary-vector backbone, thus amplification of its DNA fragment is considered as positive control.
