# Supplementary material for: The molecular basis for allelic differences suggests Restorer-of-fertility 1 is a complex locus in sugar beet (Beta vulgaris L.)
Source: BMC Plant Biol. 2020 Nov 3;20:503. doi: 10.1186/s12870-020-02721-9 (PMC7607634; doi:10.1186/s12870-020-02721-9)
Supplement: Supplementary file 6 — Additional file 6: Figure S4. Scatter plot of quantity of mRNA associated with the generation of 200-kDa complex and difference in the 250-kDa complex accumulation from rf1rf1. Positive correlation between the amount of mRNA of 200 kDa generatives and Δ250kDa in meiosis (actin) and tetrad (actin and ef1α) stages is shown. [file 12870_2020_2721_MOESM6_ESM.pptx]

## Slide 1
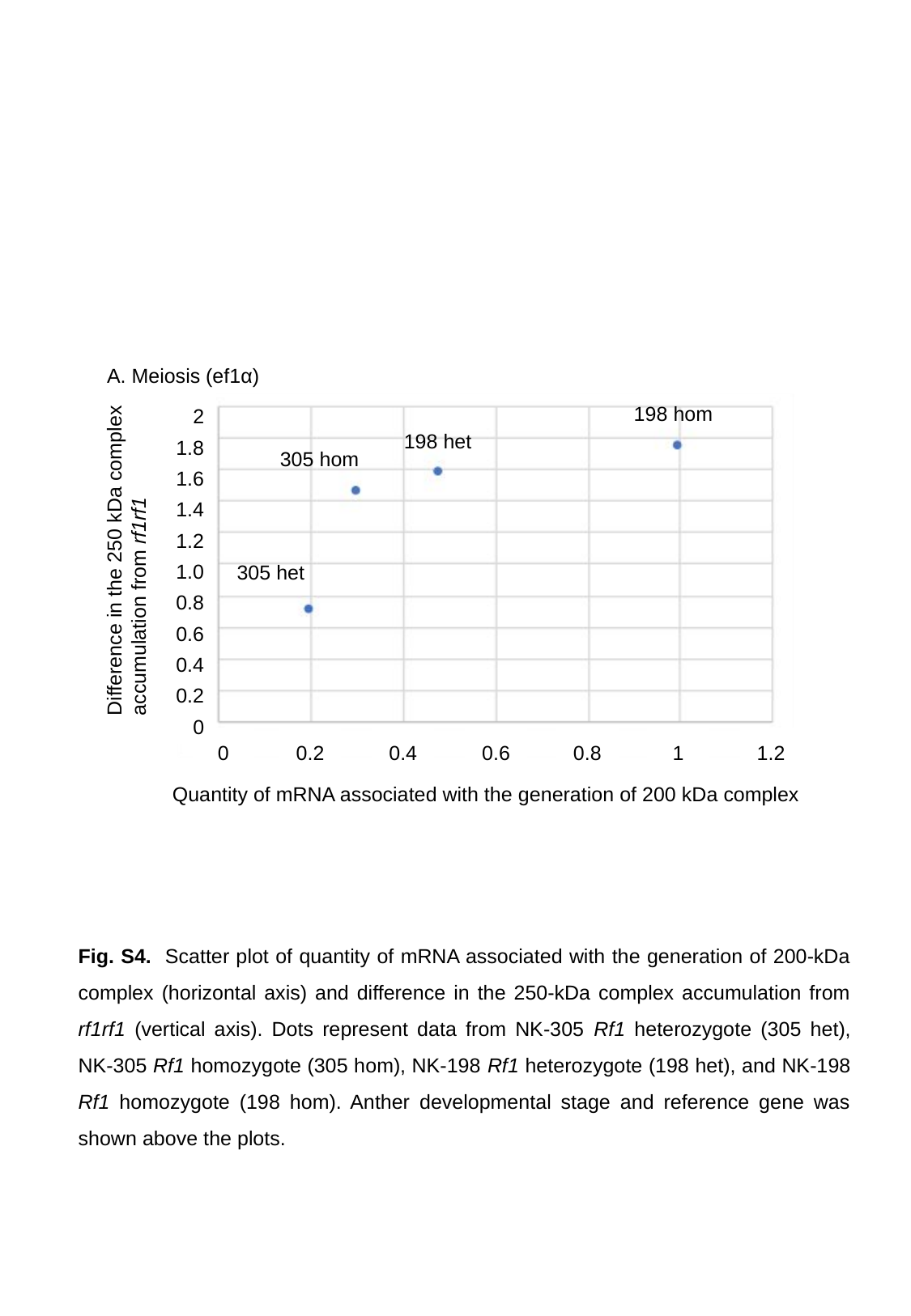

A. Meiosis (ef1α)
198 hom
2
1.8
1.6
1.4
1.2
1.0
0.8
0.6
0.4
0.2
0
198 het
305 hom
Difference in the 250 kDa complex accumulation from rf1rf1
305 het
1.2
0
0.2
0.4
0.6
0.8
1
Quantity of mRNA associated with the generation of 200 kDa complex
Fig. S4. Scatter plot of quantity of mRNA associated with the generation of 200-kDa complex (horizontal axis) and difference in the 250-kDa complex accumulation from rf1rf1 (vertical axis). Dots represent data from NK-305 Rf1 heterozygote (305 het), NK-305 Rf1 homozygote (305 hom), NK-198 Rf1 heterozygote (198 het), and NK-198 Rf1 homozygote (198 hom). Anther developmental stage and reference gene was shown above the plots.

## Slide 2
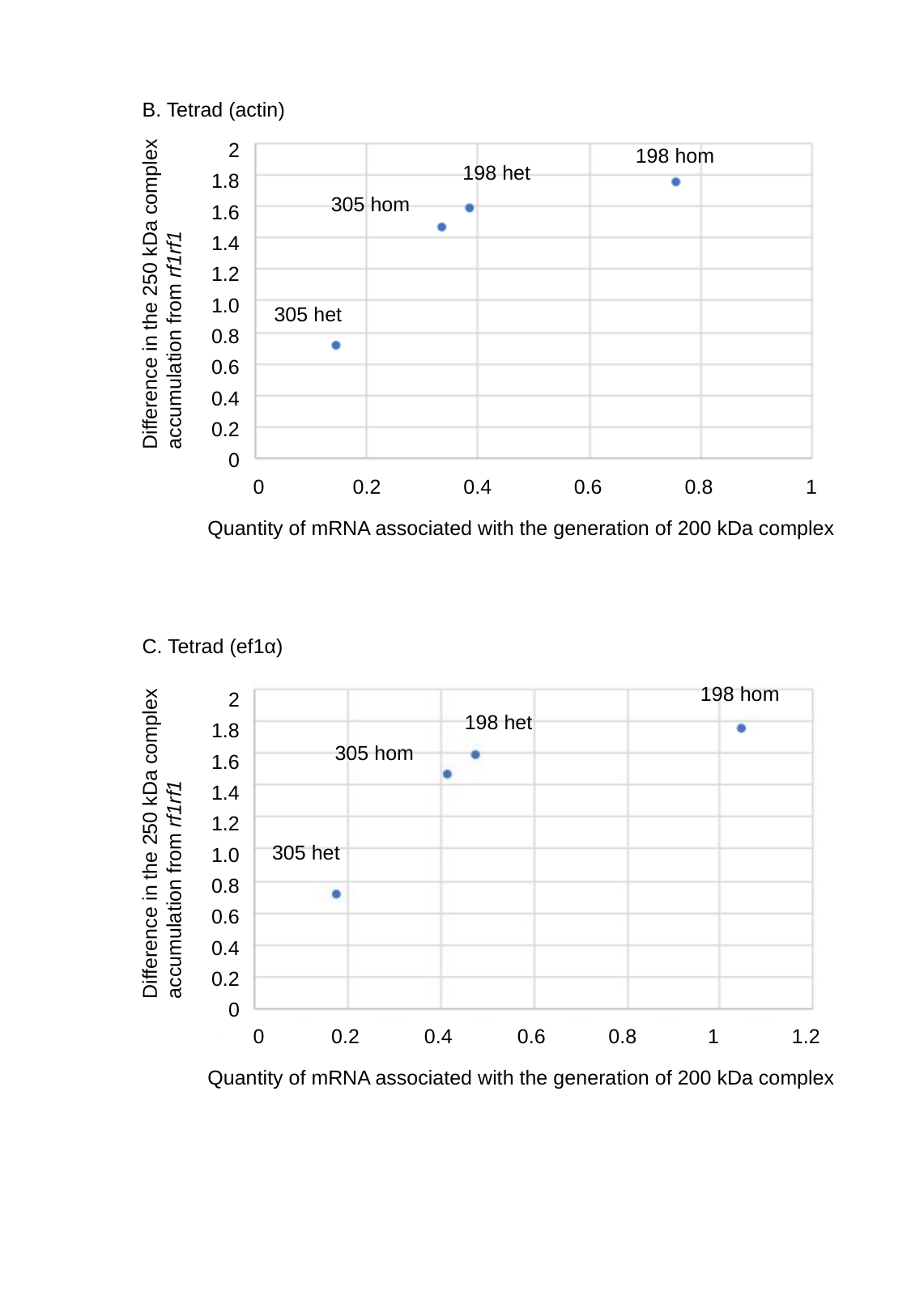

B. Tetrad (actin)
2
1.8
1.6
1.4
1.2
1.0
0.8
0.6
0.4
0.2
0
198 hom
198 het
305 hom
Difference in the 250 kDa complex accumulation from rf1rf1
305 het
0
0.2
0.4
0.6
0.8
1
Quantity of mRNA associated with the generation of 200 kDa complex
C. Tetrad (ef1α)
198 hom
2
1.8
1.6
1.4
1.2
1.0
0.8
0.6
0.4
0.2
0
198 het
305 hom
Difference in the 250 kDa complex accumulation from rf1rf1
305 het
1.2
0
0.2
0.4
0.6
0.8
1
Quantity of mRNA associated with the generation of 200 kDa complex
