# Supplementary material for: The molecular basis for allelic differences suggests Restorer-of-fertility 1 is a complex locus in sugar beet (Beta vulgaris L.)
Source: BMC Plant Biol. 2020 Nov 3;20:503. doi: 10.1186/s12870-020-02721-9 (PMC7607634; doi:10.1186/s12870-020-02721-9)
Supplement: Supplementary file 8 — Additional file 8. Uncropped images. Uncropped images used for Figs. 2, 3 and 4 are shown. [file 12870_2020_2721_MOESM8_ESM.pptx]

## Slide 1
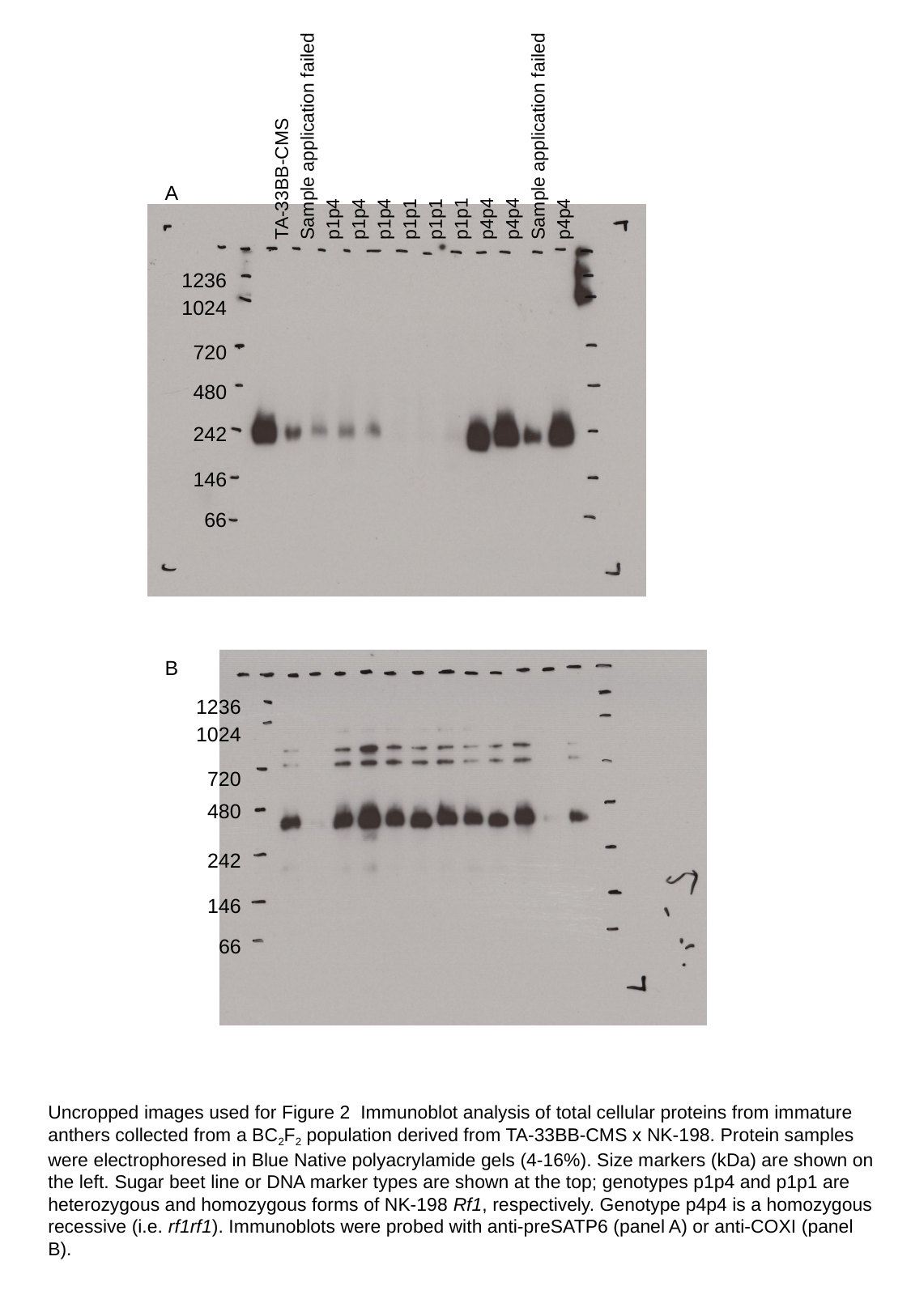

TA-33BB-CMS
Sample application failed
p1p4
p1p4
p1p4
p1p1
p1p1
p1p1
p4p4
p4p4
Sample application failed
p4p4
A
1236
1024
720
480
242
146
66
B
1236
1024
720
480
242
146
66
Uncropped images used for Figure 2 Immunoblot analysis of total cellular proteins from immature anthers collected from a BC2F2 population derived from TA-33BB-CMS x NK-198. Protein samples were electrophoresed in Blue Native polyacrylamide gels (4-16%). Size markers (kDa) are shown on the left. Sugar beet line or DNA marker types are shown at the top; genotypes p1p4 and p1p1 are heterozygous and homozygous forms of NK-198 Rf1, respectively. Genotype p4p4 is a homozygous recessive (i.e. rf1rf1). Immunoblots were probed with anti-preSATP6 (panel A) or anti-COXI (panel B).

## Slide 2
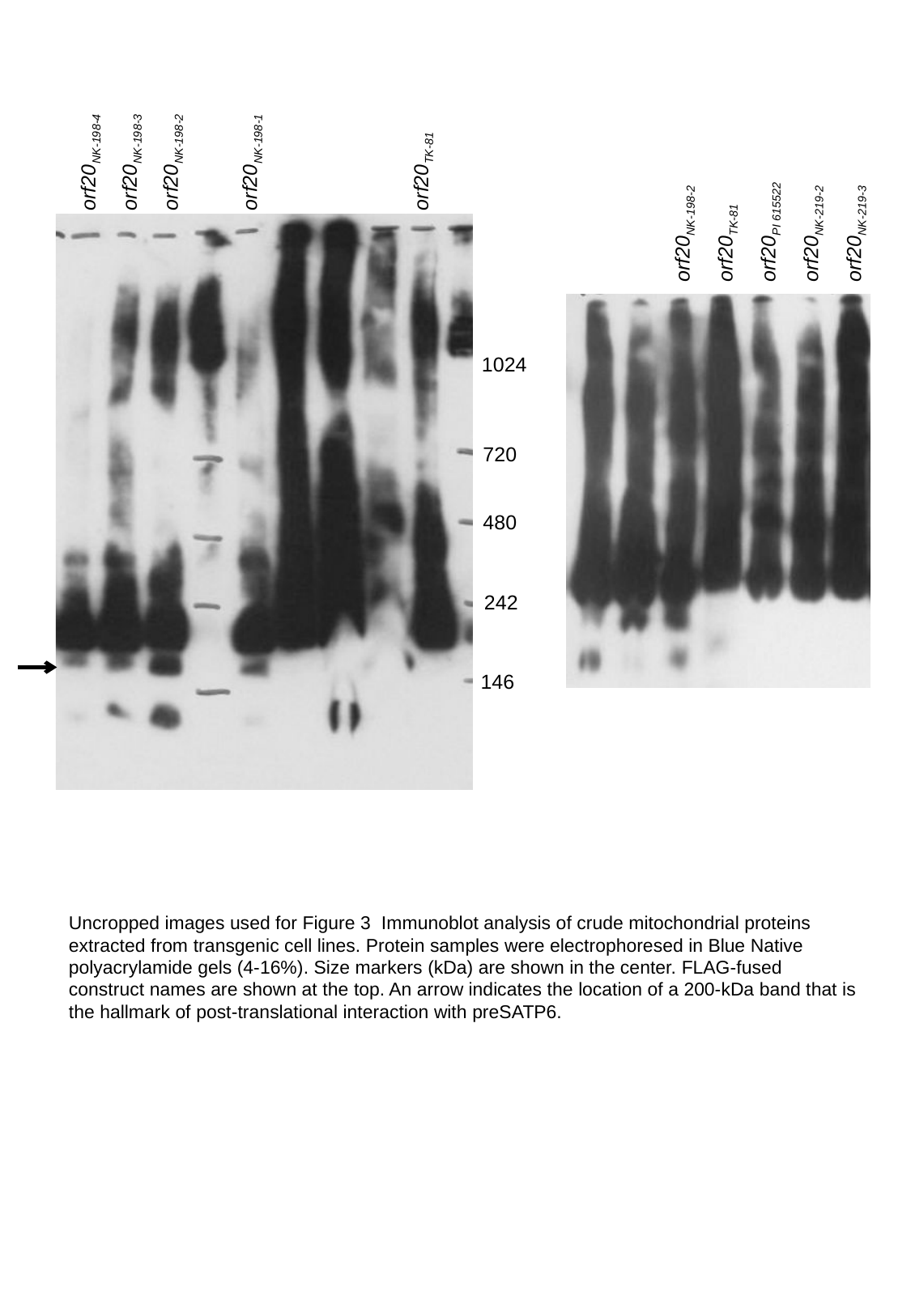

orf20NK-198-4
orf20NK-198-3
orf20NK-198-2
orf20NK-198-2
orf20TK-81
orf20PI 615522
orf20NK-219-2
orf20NK-219-3
orf20NK-198-1
orf20TK-81
1024
720
480
242
146
Uncropped images used for Figure 3 Immunoblot analysis of crude mitochondrial proteins extracted from transgenic cell lines. Protein samples were electrophoresed in Blue Native polyacrylamide gels (4-16%). Size markers (kDa) are shown in the center. FLAG-fused construct names are shown at the top. An arrow indicates the location of a 200-kDa band that is the hallmark of post-translational interaction with preSATP6.

## Slide 3
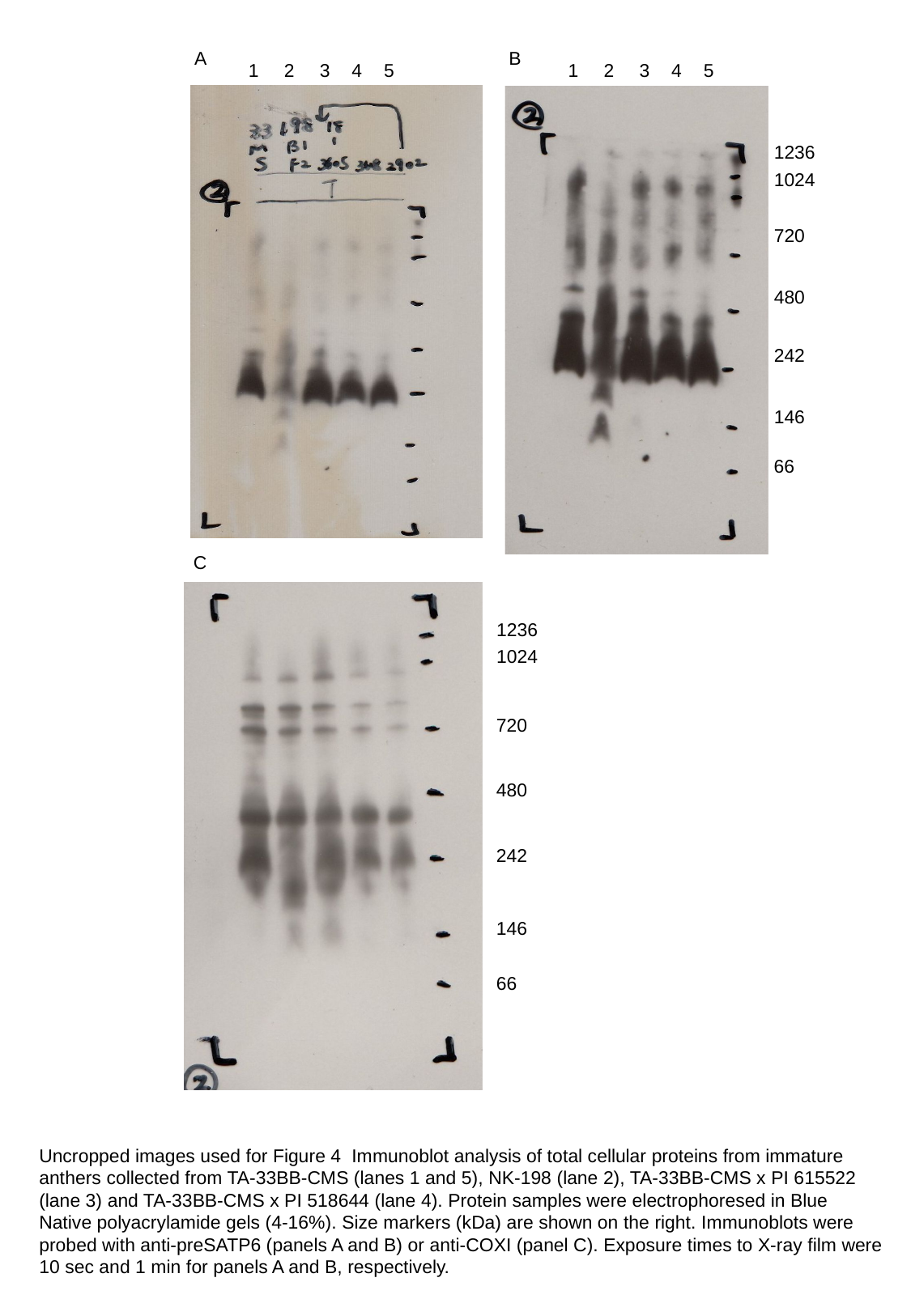

B
A
1
2
3
4
5
1
2
3
4
5
1236
1024
720
480
242
146
66
C
1236
1024
720
480
242
146
66
Uncropped images used for Figure 4 Immunoblot analysis of total cellular proteins from immature anthers collected from TA-33BB-CMS (lanes 1 and 5), NK-198 (lane 2), TA-33BB-CMS x PI 615522 (lane 3) and TA-33BB-CMS x PI 518644 (lane 4). Protein samples were electrophoresed in Blue Native polyacrylamide gels (4-16%). Size markers (kDa) are shown on the right. Immunoblots were probed with anti-preSATP6 (panels A and B) or anti-COXI (panel C). Exposure times to X-ray film were 10 sec and 1 min for panels A and B, respectively.
